# The role of reticulophagy under early phase phosphate starvation in plant cells

**DOI:** 10.1080/27694127.2022.2089511

**Published:** 2022-06-19

**Authors:** Yushi Yoshitake, Kohki Yoshimoto

**Affiliations:** Department of Life Sciences, School of Agriculture, Meiji University, 1-1-1, Tama-ku, Kawasaki-shi, Kanagawa 214-8571, Japan

**Keywords:** Early phosphate starvation, reticulophagy, ER stress, iron, lipid-ROS

## Abstract

Inorganic phosphate (Pi) is one of the most important nutrients for plant growth. Under Pi starvation, intracellular components are degraded for Pi recycling in plant cells. Macroautophagy/autophagy is a process for vacuolar degradation of cytoplasmic components including organelles, but it is still unclear whether this process is involved in plant growth and Pi recycling during Pi starvation. Recently, we reported that the degradation of endoplasmic reticulum (ER) by selective autophagy, termed reticulophagy, contributes to Pi recycling and is an important stress response in the early phase of Pi starvation. During this phase, oxidized lipids are accumulated in the plant cell in an iron ion-dependent manner, and this accumulation causes ER stress which induces reticulophagy. As a result, the Pi contents are maintained at a sufficient level during early Pi starvation, suppressing any late Pi starvation responses, such as membrane lipid remodeling. Thus, we proposed that ER stress-induced reticulophagy is an important Pi salvage system during the early phase of Pi starvation in plants.

## Autophagy contributes to Pi recycling under early Pi starvation

Inorganic phosphate (Pi) is an essential nutrient for plant growth and is used to produce many fundamental biological molecules such as nucleotides, phospholipids, and adenosine triphosphate. However, Pi is often unavailable to plants because it naturally forms insoluble complexes with metals, and because soil microbes convert Pi into organic phosphate. Therefore, plants have developed several response mechanisms to overcome this frequent risk of Pi starvation. Pi recycling is one of the Pi starvation responses, and it is mediated by enzymes responsible for the degradation of intracellular components like phospholipids and nucleic acids. The endoplasmic reticulum (ER) is a good target for Pi recycling because it has a large surface area and the main component of the ER membrane is phospholipids. Under Pi starvation, phospholipases degrade the phospholipids in the ER membrane to provide Pi and diacylglycerols (DAGs). The DAGs are delivered to plastids where they are used as substrate to produce galactolipids which do not contain any Pi. These galactolipids then replace the degraded membrane phospholipids, thus maintaining the membrane function, as well as enabling the Pi from the membrane phospholipids to be reused elsewhere in the cell. This process is called “membrane lipid remodeling”.

Previous studies have shown that autophagy is induced in plants under stress from deficiencies in nutrients such as carbon, nitrogen and zinc, and that this autophagy response is important for plant growth. To evaluate the involvement of autophagy in Pi recycling under Pi starvation, we measured Pi content in the leaves after transfer from Pi-sufficient (+Pi) to Pi-depleted (−Pi) conditions, and compared the temporal changes in wild-type and autophagy-defective mutants (*atg2* and *atg5*). Four days after transfer (DAT) to −Pi conditions (DAT4), the Pi content in wild type falls to less than 10% of that before transfer to −Pi[1]. Based on these data, we defined the periods before DAT3 and after DAT4 as “early” and “late” −Pi conditions, respectively. Under early −Pi conditions, although there is no visible phenotype, the Pi content is lower in *atg2* and *atg5* mutants than in the wild type, suggesting that autophagy contributes to Pi recycling under early Pi starvation [1].

## Lipid-ROS induces reticulophagy via ER stress under early Pi starvation

Next, we compared organelle behavior under early +Pi and −Pi conditions to determine the target of autophagy degradation under early Pi starvation. There is a higher number of autophagic bodies (ABs) containing ER under early −Pi conditions than under early +Pi conditions, but there are no ABs containing any other organelles, such as chloroplasts, peroxisomes or mitochondria, under either condition [1]. Therefore, the main target of autophagy induced by early −Pi conditions is ER. Interestingly, the reticulophagy is suppressed in an ER stress response mutant, *ire1a ire1b*, under early −Pi conditions, suggesting that the early Pi starvation-induced reticulophagy is regulated by the ER stress response [1].

Further analysis indicates that Fe limitation in media suppresses Pi starvation-induced reticulophagy through mitigation of ER stress [1]. We found that plants accumulate more Fe under early −Pi conditions than under early +Pi conditions [1]. It is well known that excess Fe generates hydroxyl radicals (·OH) and oxidized lipids (lipid-ROS), which induce ER stress through the Fenton reaction. To know whether this occurs under early −Pi conditions, accumulation levels of ·OH, lipid-ROS and hydroxyl fatty acids in mesophyll cells were compared in plants grown on media with or without Fe under early −Pi conditions [1]. The results indicated that under early −Pi conditions ·OH and lipid-ROS accumulate to high levels. Also, hydroxyl fatty acids accumulate in phosphatidylcholine and phosphatidylethanolamine, which are the main components of various biological membranes, except for plastids [1]. However, the accumulation of oxidized products as well as the high transcript levels of ER stress marker genes is suppressed by Fe limitation in the media [1]. Moreover, ferrostatin-1, an inhibitor of Fe-mediated lipid-ROS accumulation, suppresses reticulophagy under early −Pi conditions [1]. These data suggest that Fe-mediated lipid-ROS generation induces reticulophagy via ER stress.

## The role of reticulophagy in the response to Pi starvation in plants

Finally, we compared the ratio of membrane lipid composition in the wild type and the *ire1a ire1b* mutant, in which reticulophagy is suppressed, to assess the effect of reticulophagy on membrane lipid remodeling, a late Pi starvation response. Under early −Pi conditions, the value of the –Pi:+Pi ratio of phospholipids in the *ire1a ire1b* is less than in the wild type, whereas there is a higher value of the –Pi:+Pi ratio of galactolipids, meaning that the onset of membrane lipid remodeling is faster in the mutant [1]. These data indicate that reticulophagy suppresses the late Pi starvation response.

**Figure 1.**
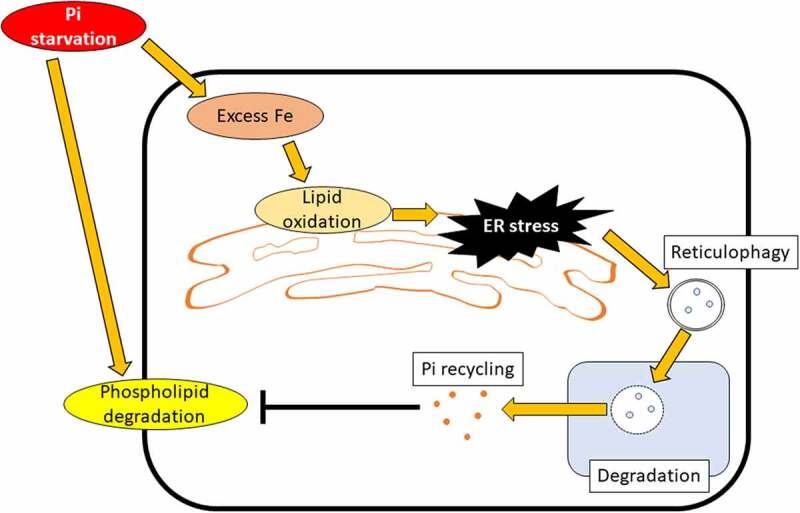
The scheme of reticulophagy under early Pi-starvation conditions. Under this condition, excess Fe oxidizes ER membrane lipids. The resulting lipid-ROS induces reticulophagy through ER stress responses to salvage Pi from the ER membrane, which suppresses late Pi starvation responses [1].

Our findings clearly suggest that ER stress-induced reticulophagy is an important Pi salvage system under early Pi starvation in plants. Plants may have two stepwise systems for salvaging Pi from intracellular components in order to effectively adapt to fluctuating Pi starvation in nature; the early Pi starvation response is reticulophagy and the late Pi starvation response is membrane lipid remodeling (Figure 1).
